# Generating circularly polarized luminescence from clusterization‐triggered emission using solid phase molecular self-assembly

**DOI:** 10.1038/s41467-021-25789-9

**Published:** 2021-09-17

**Authors:** Peilong Liao, Shihao Zang, Tongyue Wu, Hongjun Jin, Wenkai Wang, Jianbin Huang, Ben Zhong Tang, Yun Yan

**Affiliations:** 1 Beijing National Laboratory for Molecular Sciences (BNLMS), College of Chemistry and Molecular Engineering, Peking University, Beijing, 100871 China; 2 Shenzhen Institute of Molecular Aggregate Science and Engineering, School of Science and Engineering, The Chinese University of Hong Kong, 2001 Longxiang Boulevard, Longgang, Shenzhen, Guangdong 518172 China

**Keywords:** Organic molecules in materials science, Self-assembly, Colloids

## Abstract

Purely-organic clusterization‐triggered emission (CTE) has displayed promising abilities in bioimaging, chemical sensing, and multicolor luminescence. However, it remains absent in the field of circularly polarized luminescence (CPL) due to the difficulties in well-aligning the nonconventional luminogens. We report a case of CPL generated with CTE using the solid phase molecular self-assembly (SPMSA) of poly-L-lysine (PLL) and oleate ion (OL), that is, the macroscopic CPL supramolecular film self-assembled by the electrostatic complex of PLL/OL under mechanical pressure. Well-defined interface charge distribution, given by lamellar mesophases of OL ions, forces the PLL chains to fold regularly as a requirement of optimal electrostatic interactions. Further facilitated by hydrogen bonding, the through-space conjugation (TSC) of orderly aligned electron-rich O and N atoms leads to CTE-based CPL, which is capable of transferring energy to an acceptor via a Förster resonance energy transfer (FRET) process, making it possible to develop environmentally friendly and economic CPL from sustainable and renewable materials.

## Abstract (web summary)

Organic cluster-triggered emission (CTE) displays promising application in bioimaging, chemical sensing, and multicolor luminescence but circular polarize luminescence derived from CTE remains less studied. Here, the authors demonstrate CTE based CPL emission from a supramolecular film derived from solid phase molecular self-assembly.

## Introduction

It has long been recognized that the generation of fluorescence requires specific atomic or molecular structures with delocalizable electrons^1–3^. In general, these structural criteria are found in broad categories of rigid and conjugated aromatics, extended π systems, or unsaturated heterocyclic systems containing high multiplicities of heteroatoms with unoccupied p-orbitals^4,5^. However, recently a large number of molecules without conjugated groups were found to give considerable emission^4,6–8^. These molecules generally contain abundant electron-rich atoms, such as N, O, S, P, and Si. Tang and Yuan et al. revealed that when these atoms are clustered together, the extended delocalization of the lone pair electrons will occur through-space conjugation (TSC), which results in conformation rigidification and clusterization‐triggered emission (CTE)^9,10^. Since conjugated groups are not required, CTE, with the advantages of facile synthesis, good water solubility, low biological toxicity, etc.^9,11–14^, has become the potent alternative for the conventional emission in many fields, such as color-tunable light-emitting^15–20^, room-temperature phosphorescence^11–14,21–26^, as well as bioimaging and chemical sensing^13,22–24,27–30^. It would be very promising if CTE is able to replace traditional emissions in an even vast number of fields.

Circularly polarized luminescence (CPL) is attracting intensive interest in recent years, owing to its significant importance in photoelectronics^31,32^, nonlinear optics^33,34^, three-dimensional display^35^, biological probes^36–38^, and data recording^39,40^. Up to date, materials displaying CPL ability are mostly based on conventional organic emitters^41–43^, aggregation-induced emission luminogens^44^, chiral lanthanide complexes^45,46^, or clustered metal atoms^47–52^. A crucial requirement for the occurrence of CPL is the alignment of chromophores in chiral environment^41,53,54^, which is still an insurmountable challenge in a CTE system^55–57^ due to the heterogeneity of clusteroluminogens^9,10^. The absence of purely-organic CTE from the field of CPL makes it difficult to create biocompatible CPL materials economically.

Herein, we report a case of CPL generated from purely organic CTE using the strategy of solid-phase molecular self-assembly (SPMSA) proposed by us^58–60^. In this strategy, a precipitate composed of a pair of oppositely charged polyelectrolyte and amphiphile was first generated in water, and further subjected to mild mechanical pressure to facilitate the merging of nanometer-sized hydrophobic domains into mesophases. As such, a free-standing supramolecular film came into formation owing to the bridging of these mesophases with polyelectrolyte chains. Considering the hydrophobic mesophases are very alike giant 2D rigid supramolecular polymers with well-defined interface charge distribution, we hypothesize that this unique structural feature is able to induce well-defined folding of polyelectrolytes composed of chiral units, thus leads to CTE-based CPL.

**Fig. 1:**
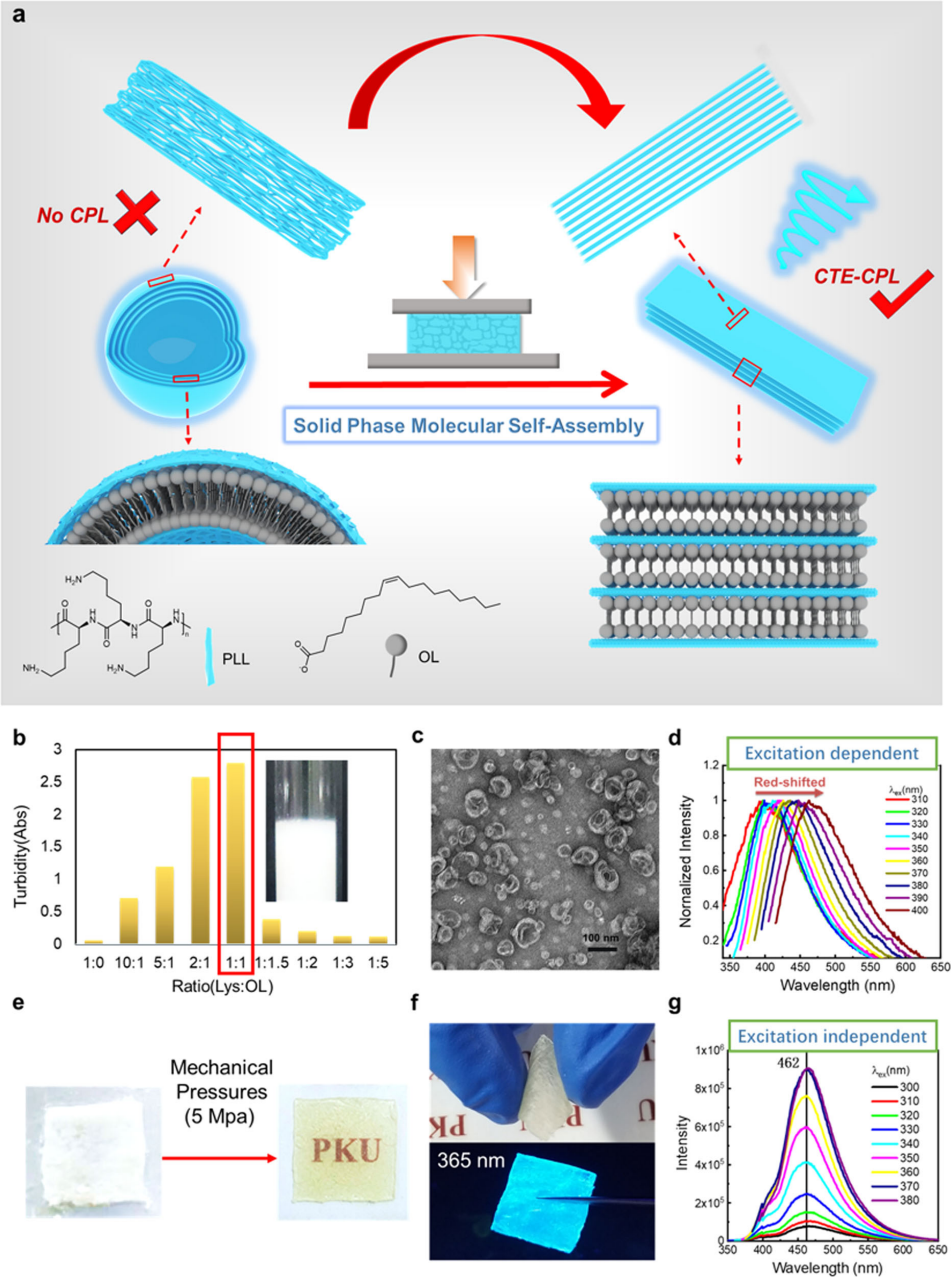
Solid phase molecular self-assembly (SPMSA) of α-poly-l-lysine-sodium oleate (PLL-OL). **a** The schematic illustration of the solid phase molecular self-assembly (SPMSA) of PLL-OL. **b** Turbidity of PLL-OL aqueous suspension at different lysine: oleate (Lys: OL) ratio ([Lys] = 10 mM). **c** TEM (transmittance electron microscopy) image for the structures formed in the PLL-OL suspension (Lys:OL = 1:1, [OL] = [Lys] = 10 mM). **d** Emission spectra of PLL-OL aqueous suspension at different excitation wavelengths (Lys:OL = 1:1, [OL] = [Lys] = 50 mM). **e** The film is obtained by pressing the precipitate. **f** The photo of the film under daylight (upper) and 365 nm UV light (lower), respectively. **g** Emission spectra of PLL-OL film at different excitation wavelengths.

For that to occur, cationic α-poly-l-lysine (PLL) and oleate ion (OL) were chosen to build supramolecular films through the strategy of SPMSA, since amino acids are well-known chiral molecules and poly (amino acids) have been verified to display CTE^14,23,24,61–64^. In an aqueous solution, the ionic assembly between PLL and OL results in cross-linked vesicles, where PLL displays characteristic CTE but CPL silent. However, the vesicle networks precipitate out upon centrifugation and transform into planar bilayers after applying a mild mechanical pressure (Fig. 1a). Up to our expectation, PLL folds into a well-defined structure on the interface of the bilayers formed by OLs, which results in the alignment of the clusteroluminogens (O and N atoms) during the process of TSC, as well as the significant CPL due to the expression of chirality of the amino groups in the CTE. Amazingly, through Förster resonance energy transfer (FRET), chirality transfer for the CTE-based CPL would occur, in analogy to conventional CPL. We envision that the current work would open a new paradigm in the study of CTE, making it possible to generate environmentally friendly CPL through an economic pathway.

## Results

### SPMSA of PLL-OL

Figure 1a shows the schematic process of the SPMSA with PLL and OL. As a nonaromatic poly (amino acid), PLL displays typical CTE character at pH 6–8, including concentration-dependent emission strength, excitation-dependent emission maximum, and silence in CPL (Supplementary Fig. 1). Transmission electron microscope (TEM) and dynamic light scattering (DLS) study (Supplementary Fig. 2) reveal that the CTE of PLL is originated from the heterogeneous clustering states of PLL chains. The sodium oleate (NaOL) also displays a weak CTE character in water because of the formation of vesicles (Supplementary Fig. 3). The electrostatic complex of PLL and oppositely charged OL in water changes the aggregation state of PLL but has no impact on the CTE nature (Supplementary Fig. 4). As the fraction of NaOL increases, the turbidity of the mixed system increases drastically and becomes whitish as the molar ratio between the lysine unit (Lys) and OL reaches 1:1 (Fig. 1b). Microscopic observation reveals that the addition of NaOL to the PLL solution leads to the formation of vesicles (Fig. 1c), and the vesicles are highly cross-linked as the concentration of NaOL reaches 50 mM (Supplementary Fig. 5). Figure 1d shows the excitation-dependent emission for the 1:1 complexed PLL/NaOL at 50/50 mM. These vesicular clusters are CPL silent (Supplementary Fig. 4c), and their CTE quantum yield (QY) at 365 nm is 4.56%. Lifetime measurements also indicate the presence of excitation-dependent emission species (Supplementary Fig. 4d). Clearly, the formation of vesicles is not helpful to obtain well-defined folding of the PLL chains, and consequently, no CPL can be detected.

The cross-linked vesicles precipitate out after centrifuging under 16100×*g*. Under mechanical pressures beyond 5 MPa, the fresh precipitates transform into a whitish film, which gradually becomes transparent within 1 h (Fig. 1e). This PLL-OL film is self-supporting and gives blue emission under 365 nm ultraviolet light (Fig. 1f). Elemental analysis (EA) reveals that the ratio of OL to Lys is around 1.4:1 (Supplementary Table 1), and the ratio measured by ^1^H-NMR is about 1.5:1 (Supplementary Fig. 6), which is close to the EA result. Strikingly, the emission maximum remains constant at 462 nm regardless of the excitation (Fig. 1g), while the emission strength increases with red-shifts of the excitation from 310 to 380 nm. This *λ*_ex_-independent CTE maximum and *λ*_ex_-dependent CTE intensity are in perfect analogy with classical fluorophores’ emission, indicating that the clustering state of the clusteroluminogens (O and N atoms) in PLL becomes homogeneous in the film (Supplementary Fig. 7). In line with this, only one *λ*_ex_-independent average fluorescence lifetime of around 5.76 ns is obtained (Supplementary Fig. 8). Meanwhile, the CTE QY is up to 5.56%, suggesting that the alignment of the O and N atoms is advantageous for the CTE.

### The ordered multilayer structure of the PLL-OL film

**Fig. 2:**
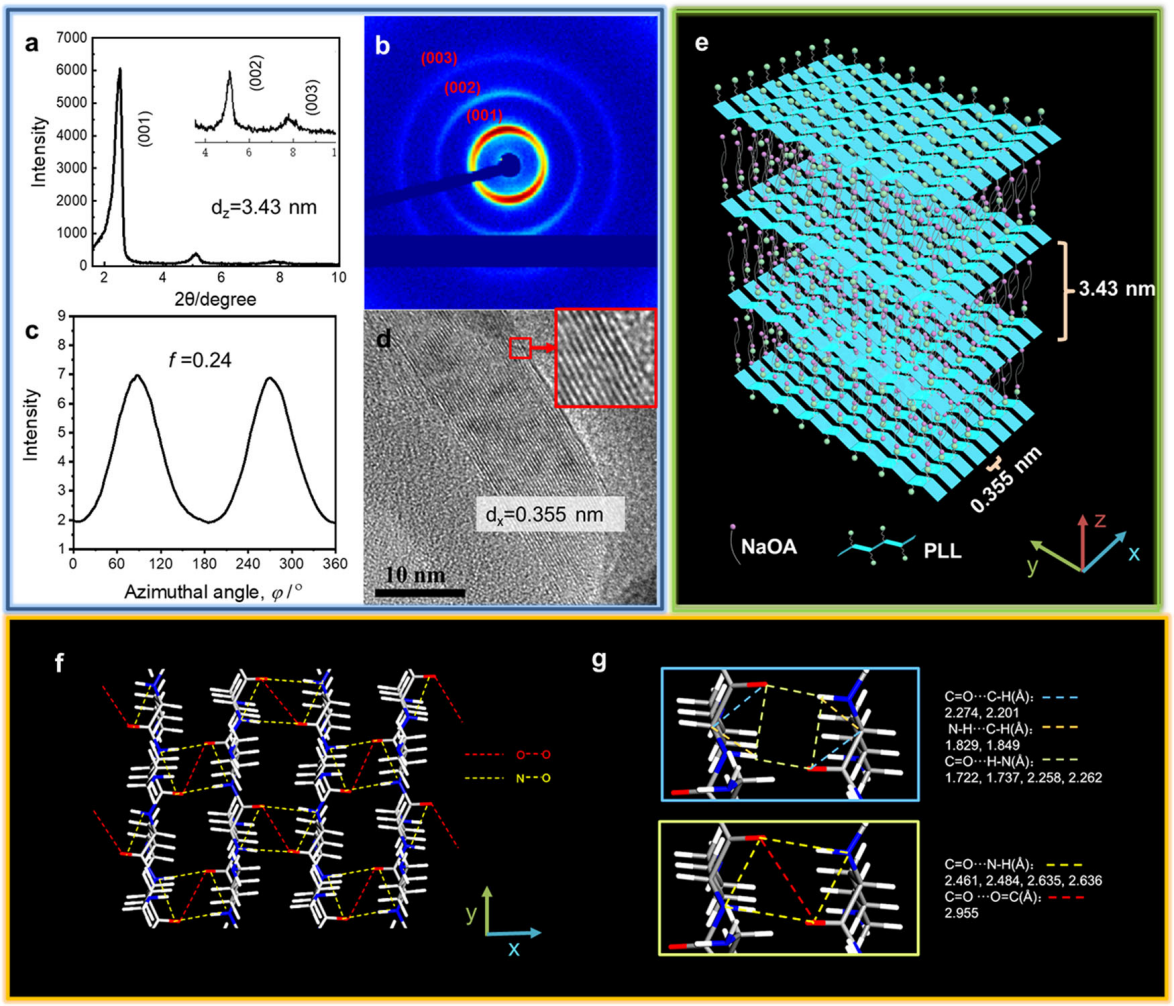
The multilayer ordered structure of the PLL-OL film. **a** XRD (X-ray diffraction) patterns, **b** 2D SAXS (2-dimensional small-angle X-ray scattering) image, **c** azimuthal angle (*φ*) plot, and **d** TEM image of the PLL-OL films. **e** Schematic illustrations of PLL-OL self-assembled multilayer ordered structure. **f** Exampled through-space electronic communications among N⋅⋅⋅O and O⋅⋅⋅O units in PLL-OL film. **g** Exampled intermolecular interaction between PLL chains.

In order to reveal the molecular packing mode in the PLL-OL film, X-ray diffraction (XRD) measurements were performed. The XRD pattern in Fig. 2a reveals 3 diffractions with a spacing ratio of 1:2:3, corresponding to the (100), (200), and (300) Miller Indices of a 2D lamellar mesostructure^65,66^. The distance between the bilayers is estimated with the Bragg equation to be 3.43 nm, which is 0.2 nm smaller than twice the extending length of the oleate molecule (Supplementary Fig. 9), indicating the presence of staggered surfactant bilayer domains in the film. Furthermore, the two-dimensional small-angle X-ray scattering (2D SAXS) pattern of the PLL-OL film shows two obvious dark arcs in the equatorial direction (Fig. 2b). The corresponding azimuthal angle (*φ*) plot features two sharp peaks at *φ* = 90° and 270°. The calculated orientation order parameter *(f*) is 0.24 (Fig. 2c), indicating that the OL bilayers are well-aligned in the film. Meanwhile, the TEM image for the cross-section of the PLL-OL film shows light and dark stripes with a spacing of 0.355 nm (Fig. 2d and Supplementary Fig. 10a). Tilted angle experiments indicate that the stripes would become more diffuse as the sample stage tilted from −5° to 10° (Supplementary Fig. 11), indicating these stripes result from the overlaid bilayers. Since the dark stripes are generated from the scattering of the electron beam by electron-rich atoms, it reveals the region of clustered O and N atoms. This indicates that the PLL chains have folded in such a way that all the O and N atoms have aligned in a row, as illustrated in Fig. 2e. It is noticed that the XRD measurements in the wide-angle region reveal a considerable broad peak at 2*θ* = 20° (Supplementary Fig. 10b). The corresponding spacing is 0.451 nm. This distance can be regarded as consistent with the 0.355 nm separation between the stripes observed under TEM. It is possible that the spacing shrinks under the electron beam in TEM. Figure 2f, g demonstrates the possible chain alignment and the TSC between the N and O atoms in neighboring chains. Owing to the electrostatic interaction between the ammonium positive charges (NH_3_^+^) of PLL side groups and the carboxylate negative charges of OL, the aligned PLL chains lay on the interface of the bilayers formed by the oleate, as shown in Fig. 2e.

### Humidity responsive CTE-based CPL

**Fig. 3:**
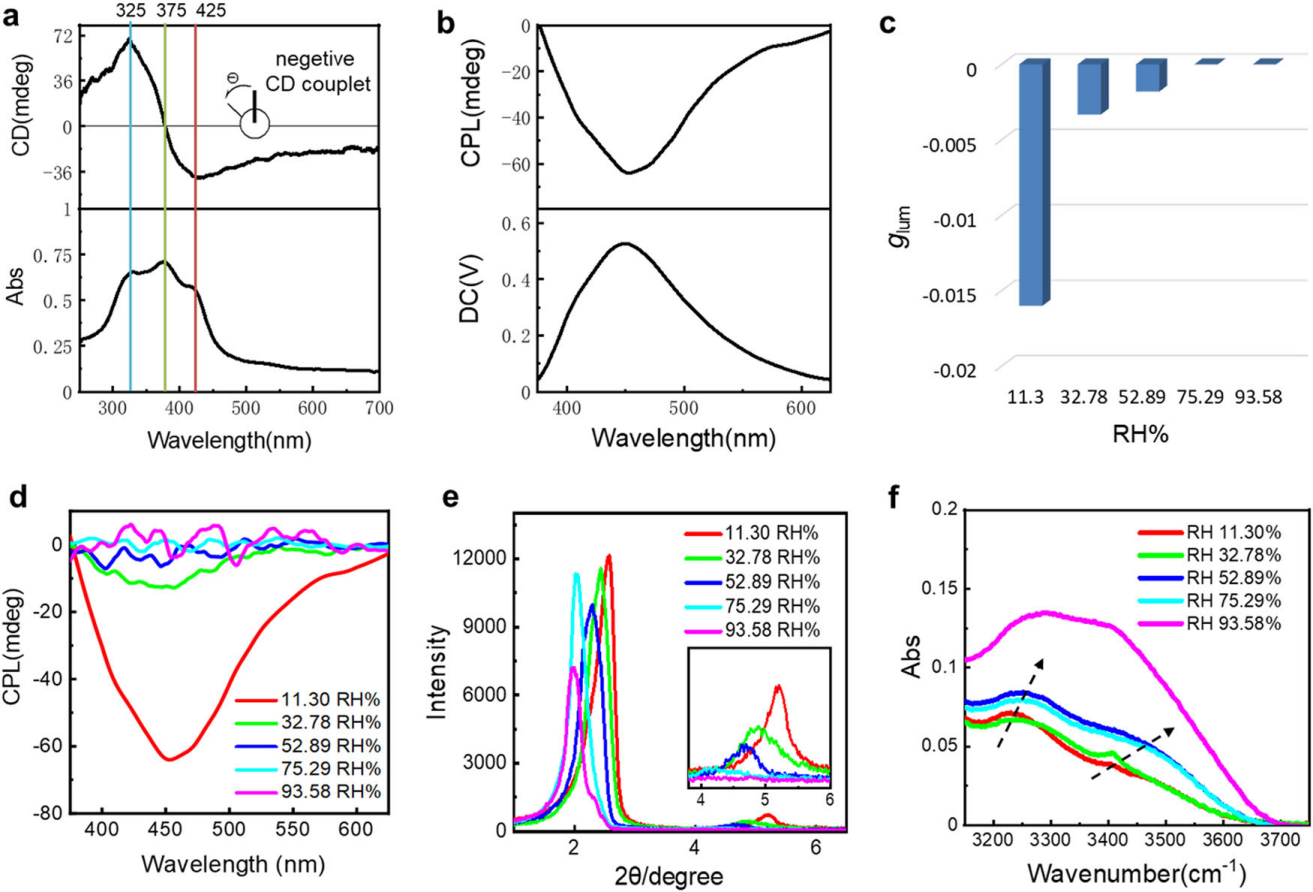
Humidity responsiveness of the clusterization triggered emission (CTE)-based circularly polarized luminescence (CPL). **a** Circular dichroism (CD) and ultraviolet–visible (UV–vis) spectra and **b** CPL spectra (*λ*_ex_ = 330 nm) of PLL-OL film (relative humidity (RH): 11.30%). **c** Emission dissymmetry factor *g*_lum_ value, **d** CPL spectra (*λ*_ex_ = 330 nm), **e** XRD patterns, and **f** FT-IR (Fourier transition-inferred) spectra of the PLL-OL film at different relative humidity.

The well-defined clusterization of PLL chains in the film has resulted in a significant chiroptical signal. Figure 3a shows that an exciton chiral signal featuring Davydov splitting^67^ occurs at 375 nm, with the first Cotton effect (Δ*ε*1, longer wavelength) value being −40 at 425 nm and the second Cotton effect (Δ*ε*2, shorter wavelength) being 69 at 325 nm. This chiroptical signal suggests that the negative exciton chirality has been generated, and the amplitude of the couplet *A* (defined as Δ*ε*1 − Δ*ε*2) and Δ*λ* (nm) value is 109 and 50, respectively. It is noticed that the circular dichroism (CD) signal is not originated from linear dichroism (LD), since the CD signal remains nearly constant at varying angle measurements, whereas the LD spectrum is angular dependent (Supplementary Fig. 12). In line with the reliable CD, right-handed CPL was observed. As shown in Fig. 3b, the maximum polarized emission wavelength of PLL-OL film is 454 nm, which coincides with the maximum emission of fluorescence (462 nm). The emission dissymmetric factor *g*_lum_ value, which is defined as 2(*I*_L_ − *I*_R_)/(*I*_L_ + *I*_R_), where *I*_L_ and *I*_R_ denoting the emission intensities of left-handed CPL (*l-*CPL) and right-handed CPL (*r-*CPL) component, respectively^68,69^, is −0.016 (Supplementary Fig. 13). This value is 10–1000 times higher than those generated from many conventional conjugated organic emitters since they often have a *g*_lum_ of 10^−3^–10^−5^^70–72^. To the best of our knowledge, this is the first purely organic CTE based CPL that has ever been reported, indicating it is very promising in creating CPL with green and sustainable materials.

The CPL intensity reduces significantly with increasing humidity. The *g*_lum_ value decreases rapidly from −0.016 at relative humidity (RH) 11.30% to −0.0033 at RH 32.78% and −0.0018 at RH 52.89% (Fig. 3c). A much higher humidity than 52.89% results in almost complete vanishment of the CPL signal (Fig. 3d), although the fluorescence intensity remains basically unchanged (Supplementary Fig. 14a). The reduction of the CPL is an indication of the reduced supramolecular chirality in the film. Supplementary Fig. 14b reveals that the CD signal decreases drastically as the humidity increases, and disappears completely as the RH exceeds 75.29%.

The XRD pattern in Fig. 3e suggests that the interlayer spacings increase from 3.43 to 4.42 nm as the RH increases from 11.30 to 93.58%. Meanwhile, the second-order diffraction peak is significantly weakened (Inset in Fig. 3e and Supplementary Table 2), manifesting a less ordered long-range stacking of the bilayers in the PLL-OL film. It is noticed that the distance of 4.42 nm is about 0.8 nm larger than twofold the extending length of the OL chains, indicating that the folding state of the PLL chains has changed drastically with increasing humidity. Thermogravimetric analysis (TGA) measurement suggests that the water content in the film increases with humidity. At the RH of 11.30%, the water content in the film is 0.9%, whereas it rises to 23.3% at the high RH of 93.58% (Supplementary Fig. 15 and Supplementary Table 2). This means that the electrostatic interactions between PLL chains and the 2D OL bilayers would be significantly weakened since the dielectric constant of water is 78.5 times larger than that in the vacuum. Fourier transform infrared (FT-IR) spectra manifest that the wavenumber of the N–H vibrational band at 3400 cm^−1^ increases with humidity (Fig. 3f and Supplementary Table 2), indicating the hydrogen bonds that drive the well-defined folding of the PLL chains have been weakened. (The peak shape under the humidity of 93.58 is indiscernible due to the interference from the broad and strong vibration bands of water at 3200–3600 cm^−1^.) This means that the PLL chains on their own can hardly fold regularly; the electrostatic interactions between the PLL chains and the OL bilayers are crucial for the forced intramolecular hydrogen bonding.

### CTE-based FRET leading to color-tunable CPL

**Fig. 4:**
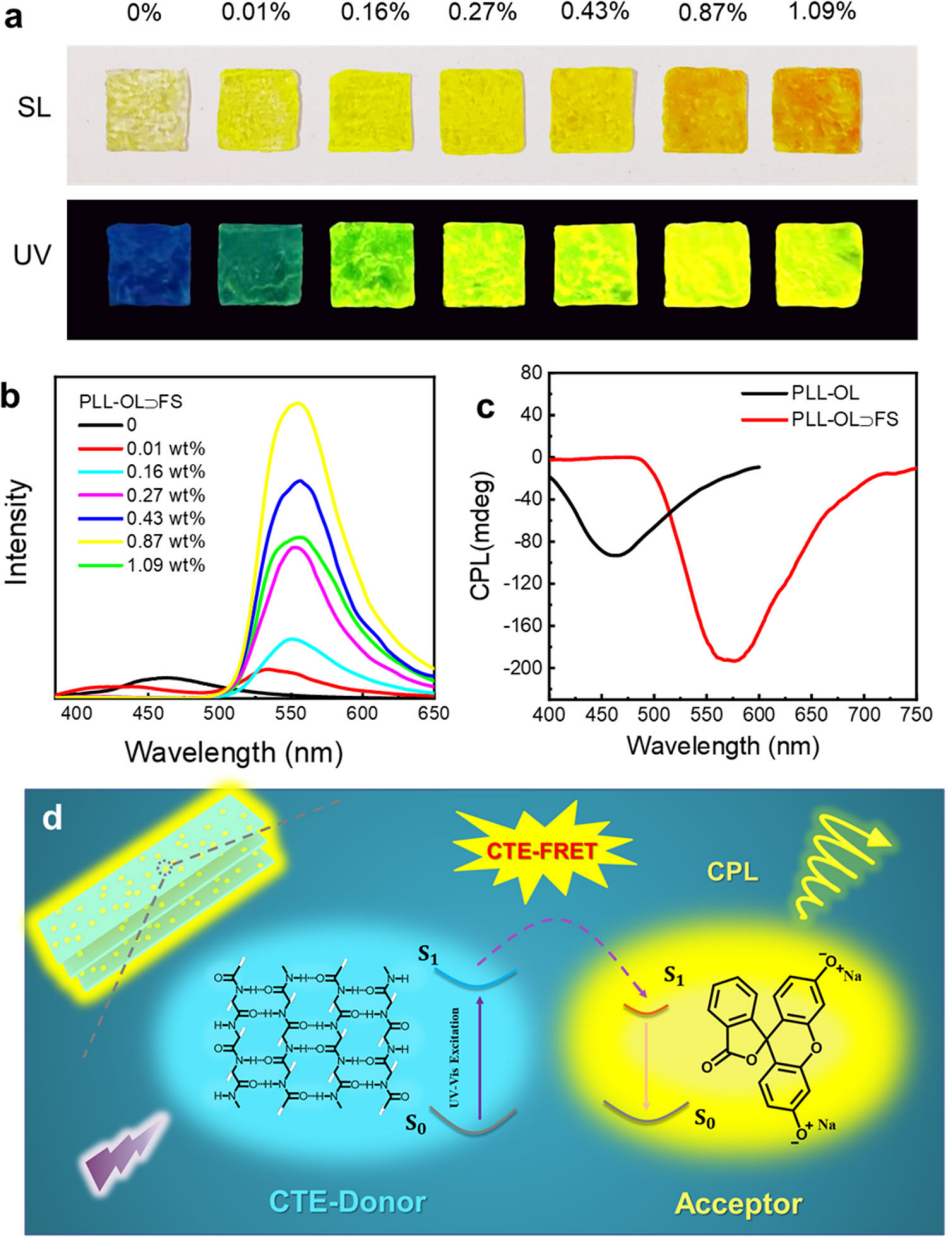
Color tunable CPL through FRET. **a** Photos and **b** emission spectra (Ex = 365 nm) of PLL-OL⊃FS films with different FS (fluorescein sodium) doping rates under daylight and 365 nm UV light. **c** CPL spectra (Ex = 342 nm, FS:0.87%) of PLL-OL vesicles and PLL-OL⊃FS film. **d** Energy transfer and the generation of CPL via the FRET (Förster resonance energy transfer) between the CTE donor of PLL-OL film and the molecular acceptor FS.

The excitation independent CTE of PLL in the PLL-OL film is very alike conventional emission generated with conjugated molecules. We then move a further step to check whether this CTE is able to undergo FRET as its conjugated counterparts do. Fluorescein sodium (FS) is a common commercial dye. The dilute solution of FS absorbs light within 400–500 nm and emits strong yellow fluorescence near 540 nm. Since the emission of PLL in the film overlaps well with the absorption of FS (Supplementary Fig. 16a), FS was doped as the energy acceptor into the film via coprecipitation. With increasing the doping amount of FS, the blue CTE of PLL decreases gradually, and the yellow emission of FS centered at 555 nm rises up (Fig. 4a, b). The maximum FS emission is observed at the FS doping rate of 0.87 wt%. Further increase of FS results in a decrease of the emission intensity, due to the notorious aggregation-caused quenching^73,74^. This means that CTE, just like conventional fluorescence, is able to undergo FRET in the presence of an energy acceptor. Supplementary Table 3 shows the changes in fluorescence lifetime, energy transfer efficiency, and absolute QY of the PLL-OL⊃FS film with different FS doping amounts. Before FS doping, the average fluorescence lifetime *τ*_*D*_ of PLL in the film is approximately 5.68 ns (Supplementary Fig. 16b and Supplementary Table 3). In the case of 0.87 wt% FS doping, the CTE lifetime of PLL in the film decreases to 2.39 ns. Based on these lifetime variations, the efficiency of energy transfer from the folded PLL to FS is estimated to be 57.9%. It is noticed that in this FRET process, the absolute QY of the system has been amplified from 5.56 to 21.9%. This enhanced QY is probably caused by the direct excitation of FS under 365 nm since the absorption of FS extends to this wavelength (Supplementary Fig. 17). Most strikingly, the supramolecular chirality of PLL is also transferred to FS, leading to a broad negative CD signal in the range of 300–500 nm, with a sharp peak occurred at 521 nm (Supplementary Fig. 18). Meanwhile, a bright yellow right-handed CPL was observed at 575 nm (Fig. 4c), and the *g*_lum_ of the maximum emission is −0.018 (Supplementary Fig. 19). This means that FS has complexed efficiently with the folded PLL chains. Based on the above experimental results, a possible energy transfer mechanism is proposed in Fig. 4d. Through the process of FRET, we can obtain color-tunable CPL simply by varying the doping ratio of the acceptors. This greatly broadens the application versatility of CTE, endowing it all the functions of traditional emissions.

## Discussion

The chain folding state of PLL is very critical for the generation of CTE-based CPL. Here, the desired chain folding of PLL can be induced by the regularly arranged negative charges on the 2D interface of the OL bilayers. Because of the electrostatic interactions, the quarternized amide groups in the PLL chains are forced to align according to the negative charge density on the 2D interface. Meanwhile, these quarternized amide groups would form hydrogen bonds with other O and N atoms, which are in the form of TSC. In this way, the clusteroluminogens are aligned on the interface of the 2D OL bilayers.

With increasing the environmental humidity, the physically adsorbed water would bond preferably with the ionic groups of PLL and OL^58^, which weakens the electrostatic interaction between them. As a result, the PLL chains would swell, and the hydrogen bonding between the clusteroluminogens is destroyed. Consequently, the N and O atoms forming TSC could not be aligned anymore, resulting in the vanishing of the supramolecular chirality. Because the electrostatic interaction in the presence of water would always be weakened, it is hard to obtain CPL in aqueous media via electrostatic interactions. That’s why the PLL/OL vesicles do not display CPL. In this sense, SPMSA would be very efficient in generating CTE-based CPL, and the CPL can be manipulated with environmental humidity. Since the acquiring and loss of water via humidity change is reversible, the CTE-based CPL can thus be reversibly switched on and off by controlling the environmental humidity, rendering it an efficient approach to create smart CPL materials.

It should be clarified that the CTE in the current study is not from artifacts aroused from impurities in the used chemicals. As the purity and the sources of both PLL and NaOL were varied, the CTE phenomenon always occurred (Supplementary Fig. 20). The NaOL powders on their own display CTE as well, since the COO^−^ groups may pack closely as a result the formation of NaOL bilayers. However, this CTE is excitation wavelength-independent, since the “clustering” state of the COO^−^ groups are uniform in the bilayers. In contrast, the CTE of PLL powders from all sources are excitation wavelength-dependent, indicating the clustering states of PLL on its own are heterogeneous (Supplementary Fig. 21). It is the regular charge density on the 2D surface of OL bilayers that forced the PLL chains to fold in a regular state. However, only when the PLL chains are folded in a certain state can CPL be generated. Actually, as the structure of PLL is changed, such as from α-PLL used in this study to ε-PLL, which is a PLL with its polymerization site different from that of a-PLL, no CPL can be obtained in all cases (Supplementary Fig. 22).

In summary, we made the purely organic CTE-based CPL with the strategy of SPMSA. The CTE material PLL was forced to align well on the interface of the self-assembled oleate bilayers so that the CTE molecule PLL not only clustered but also formed a supramolecular structure and generated 2D supramolecular exciton chirality. By controlling the folding state of PLL with environmental humidity, the supramolecular chirality status of PLL can be manipulated on and off. The emission maximum of CTE in the SPMSA is excitation independent, making it in analogy to conventional luminescence. In this way, the CTE-based CPL can be further transferred and magnified via a FRET process. We believe that the SPMSA can be an efficient strategy to transform CTE molecules from random clusters into regular arranged superstructures, making it possible to generate CPL from economic and sustainable CTE.

## Methods

### SPMSA of PLL-OL

α-PLL, with a purity of 95%, was purchased from BYD Pharmaceuticals. NaOL, chemically pure, was purchased from Sinopharm Group. The experimental water was ultra-pure water (Milli-Q water) with a resistivity of 18.2 MΩ cm obtained by a Millipore ultra-pure water machine. An aqueous solution of PLL and an aqueous solution of NaOL was added, reaching final concentrations of 50 mM for the ammonium positive charges of PLL and 50 mM for the carboxylate negative charges of NaOL. White precipitates were immediately formed after mixing and separated from the suspension by centrifugation with a speed of 16,100 × *g* for 15 min. The collected precipitates were then treated in two parallel ways: subjected to a pressure imposed by finger pressing and noodle machine manufacturing under an ambient environment.

### Humidity control experiment

The newly prepared films were suspended in an atmosphere and equilibrated with different saturated salt solutions for a week. The RH of the saturated salt solution is as follows: LiCl—11.30%, MgCl_2_—32.78%, Mg(NO_3_)_2_—52.89%, NaCl—75.29%, KNO_3_—93.58%.

### Dyes doping

After leaving the newly prepared PLL-NaOL film for 12 h, each film was weighed and cut to 0.056 g, and then 1.3 × 10^−5^ to 1.3 × 10^−1^ mol/L FS aqueous solution 1–10 μl was gradually added. The PLL-NaOL⊃FS film was pressed again with different FS mass fractions.

### Estimation of the orientation order

The orientation order parameter (*f*) was estimated using the azimuthal angle plots obtained from the 2D SAXS images^75^. The *f* values range between 0 and 1, where the former corresponds to an isotropic structure, and the latter corresponds to perfect orientation along the director. A Maier–Saupe distribution function was used to fit the azimuthal angle plots

1 $$I={I}_{0}+A\,{{{{{\rm{exp }}}}}}\,[{{\upomega }}\,{{{{{{\rm{cos }}}}}}}^{2}(\varphi -{\varphi }_{0})]$$

where *I*_0_ denotes the free baseline intensity, *φ*_0_ is the azimuth at the position of the maximal intensity, *φ* is the azimuth and *ω* is the parameter that determines the width of the distribution. After the curve fitting of this function to the azimuthal angle plot, parameters *I*_0_, *A*, and *ω* were obtained. The orientation order parameter f was determined using the following equation

2 $$f=\frac{{\int }_{-1}^{1}{P}_{2}({{{{{\rm{cos }}}}}}\,\varphi )\,{{{{{\rm{exp }}}}}}({{\upomega }}\,{{{{{{\rm{cos }}}}}}}^{2}\varphi )\,{{{{{\rm{d}}}}}}{{{{{\rm{cos}}}}}}\,\varphi }{{\int }_{-1}^{1}{{{{{\rm{exp }}}}}}\,({{\upomega }}\,{{{{{{\rm{cos }}}}}}}^{2}\varphi )\,{{{{{\rm{d}}}}}}{{{{{\rm{cos}}}}}}\,\varphi }$$

where the function *P*_2_(cos*φ*) is the second-order Legendre polynomial of cos*φ*, often referred to as the Hermans orientation function

3 $${P}_{2}\left({{{{{\rm{cos }}}}}}\,\varphi \right)=\frac{1}{2}(3\,{{{{{{\rm{cos }}}}}}}^{2}\varphi -1)$$

### Estimation of the efficiency of the transfer

The rate of energy transfer is inversely proportional to the sixth power of the distance, *r*, between the donor and acceptor. The efficiency of energy transfer *E*_FRET_ is defined with respect to *r* and *R*_o_, the characteristic Förster distance^76^ for the donor and acceptor pair by

4 $${E}_{{{{{\rm{FRET}}}}}}=\frac{1}{1+{\left(\frac{r}{{R}_{0}}\right)}^{6}}$$

where *R*_0_ is a characteristic distance for the donor–acceptor combination at which *E*_FRET_ takes the value 0.5. Experimentally, FRET can be detected in several ways^77–81^. Energy transfer causes quenching of donor fluorescence and sensitized fluorescence of the acceptor. It also reduces the donor lifetime and decreases the rate of irreversible photobleaching of the donor. In this work, experimental FRET efficiencies (*E*) were obtained from steady-state measurements using

5 $$E=1-\frac{{\tau }_{{DA}}}{{\tau }_{D}}$$

where *τ*_DA_ and *τ*_*D*_ are the measured donor fluorescence lifetimes in the absence and in presence of the acceptor, respectively.

## Supplementary information

Supplementary Information

## Data Availability

All the data and methods are present in the main text and the supplementary materials. Any other relevant data are available from the authors upon reasonable request.
