# Supplementary material for: Generating circularly polarized luminescence from clusterization‐triggered emission using solid phase molecular self-assembly
Source: Nat Commun. 2021 Sep 17;12:5496. doi: 10.1038/s41467-021-25789-9 (PMC8448880; doi:10.1038/s41467-021-25789-9)
Supplement: Supplementary file 1 — Supplementary Information [file 41467_2021_25789_MOESM1_ESM.pdf]

Supporting Information

for

Generating Circularly Polarized Luminescence from Clusterization-Triggered Emission Using Solid Phase Molecular Self-Assembly

Peilong Liao[1], Shihao Zang[1], Tongyue Wu[1], Hongjun Jin[1], Wenkai Wang[1], Jianbin Huang[1], Ben Zhong Tang[2,], Yun Yan[1,*]*

Table of Contents

1.Supplementary Methods 2

2.Supplementary Disscussion 3

2.1 The CTE characteristics of α-poly-L-lysine (PLL) aqueous solution and sodium oleate (NaOL) 3

2.2 The CTE characteristics of PLL-OL aqueous solutions 5

2.3 The composition and structure characterization of PLL-OL film 7

2.4 Supplementary for the humidity responsive CTE-based CPL 12

2.5 Supplementary for the Color tunable CPL through FRET 14

2.6 Control experiment 17

1.Supplementary Methods

X-ray diffraction (XRD) measurements were performed using a Rigaku Dmax-2400 diffractometer with Cu Kα radiation. The solid samples were placed on clean glass slides for small-angle range tests. Small angle X-ray scattering (SAXS) and wide-angle X-ray scattering (WAXS) measurements were performed using ganesha small angle x-ray scattering system of SAXSLAB company with Cu Kα radiation. Thermogravimetric analysis (TGA) experiments were carried out under nitrogen flow on TA Instrument Q600 SDT at a heating rate of 10 °C/min. Elemental Analysis (EA) of the PLL-OL film were carried out on Vario EL elemental analyzer with 0.1% accuracy for C, H and N. ^{1}H-NMR experiments were conducted on a Bruker ARX 500 MHz spectrometer while the PLL-NaOL film, PLL powder and NaOL powder were dissolved in CD_3OD.The fluorescence spectrum, fluorescence lifetime, and quantum yield of liquid samples were all given by the Edinburgh Instruments Limited FLS980 steady-state transient fluorescence / phosphorescence spectrometer (77-500K). Liquid samples can be directly measured in a quartz cell. UV-Vis spectrums were given by Shimadzu UV-1800 spectrometer. The Transmission electron microscope (TEM) photos of the samples were taken on a JEM-2100F transmission electron microscope. The acceleration voltage was 200 kV. Film samples were prepared by resin-embedded sections. The dynamic light scattering (DLS) spectrum were given by Brookhaven's Nano DLS highly sensitive particle size analyzer. The sample was filtered through a 450 nm filter before test, and the experimental results are processed by volume distribution. Circular dichroism (CD) spectra were recorded by a JASCO J-1500

spectrometer at range of 250-700 nm with scanning speed of 200 nm/min, response time of 1 s, and bandwidth of 3 nm. Circularly polarized emission (CPL) spectra were recorded by CPL-200 spectrometer at range of 400-700 nm with the HT of the Excitation light source of 600-800 V, scanning speed of 100 nm/min, response time of 2 s. The glum value is calculated after reasonable smoothing and baseline correction. And the fourier transform infrared (FT-IR) spectrums were given by NICOLET IS50 Fourier transform infrared spectrometer through attenuated total reflection (ATR).

2.Supplementary Disscussion

2.1 The CTE characteristics of α-poly-L-lysine (PLL) aqueous solution and sodium oleate (NaOL)

PLL is a typical clusterization-triggered emission (CTE) luminescent material with characteristic concentration- dependent luminescence, wave-dependent luminescence and silence in CPL, which is attributed to its heterogeneous micelle aggregation structure in solution. the fluorescence quantum yield at 0.090 M is 4.24%. (Supplementary Figure 1 and Supplementary Figure 2 respectively support this conclusion in terms of fluorescence properties and aggregate structure)

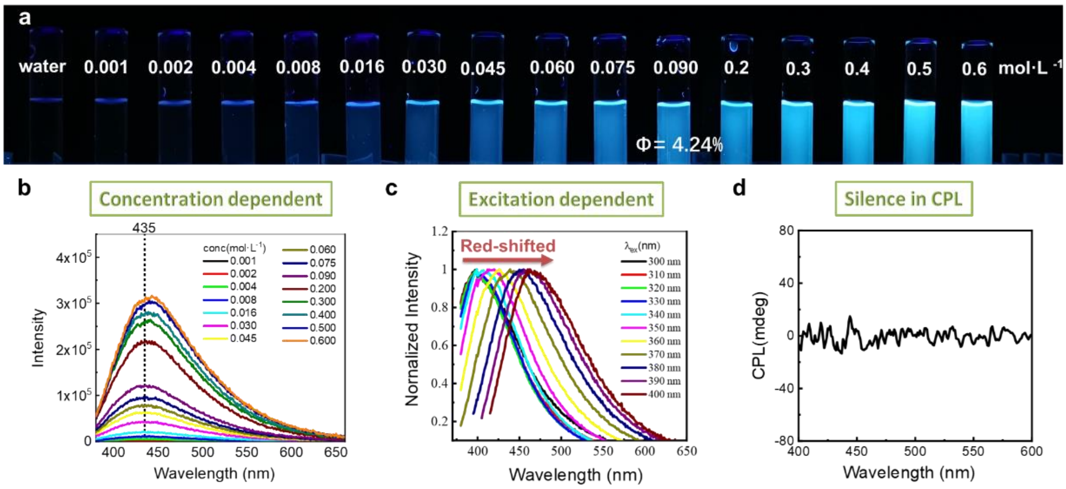

Supplementary Figure 1. The Fluorescence properties of PLL aqueous solutions. a Photographs taken under 365 nm UV light and **b** emission spectra (λ_{ex} = 365 nm) of PLL aqueous solutions at different concentrations. **c** Emission spectra of PLL (0.1 M) at different excitation wavelength. **d** CPL spectra (λ_{ex} = 350 nm) of PLL (0.1 M) aqueous solutions.

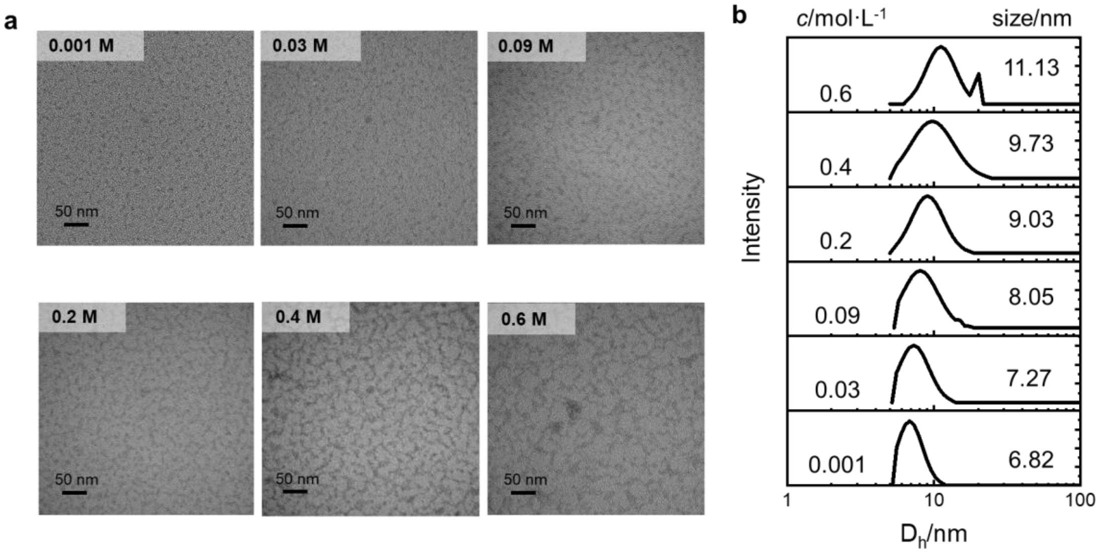

Supplementary Figure 2. The aggregate structure of PLL aqueous solutions. a TEM and **b** DLS spectra of 0.001-0.6 M PLL aqueous solutions.

The sodium oleate (NaOL) aqueous also shows typical CTE, the fluorescence quantum yield at 100 mM is 1.99%. Its critical micelle concentration (cmc) is about $1\sim2\times10^{-3}$ $mol\cdot L^{-1}$. At 0.05 $mol\cdot L^{-1}$, 50-100 nm vesicles and micron-sized large vesicles can be observed. (Supplementary Figure 3)

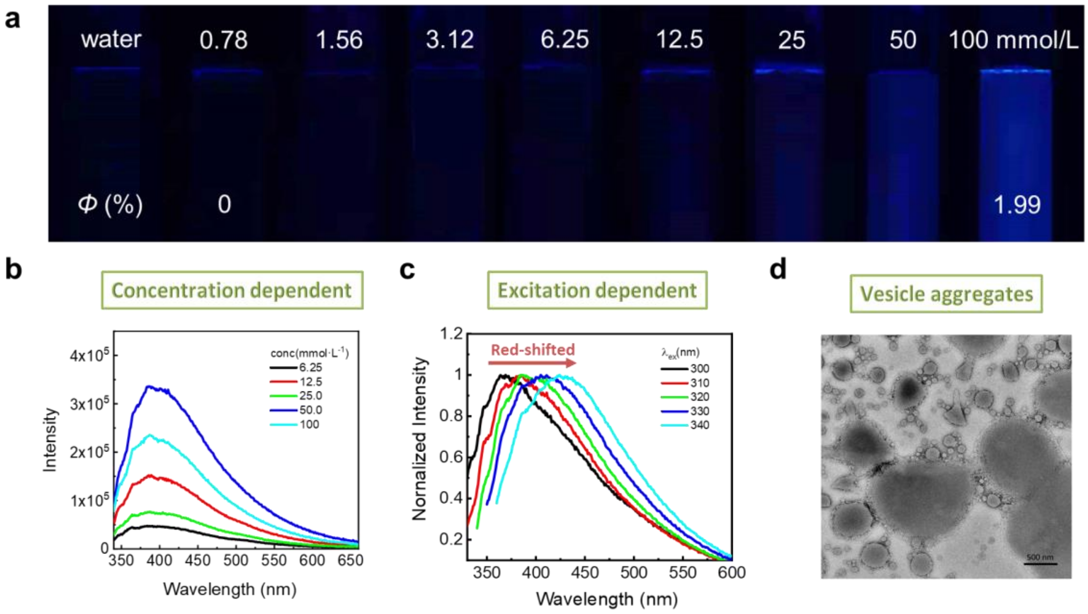

Supplementary Figure 3. The Fluorescence properties and aggregate structure of NaOL aqueous solutions. a Photographs taken under 365 nm UV light and **b** emission spectra (λ_{ex} = 365 nm) of NaOL aqueous solutions at different concentrations. **c** Emission spectra of NaOL (0.1 M) at different excitation wavelength. **d** TEM image of NaOL (50 mM) aqueous solutions.

2.2 The CTE characteristics of PLL-OL aqueous solutions

PLL-OL aqueous solutions at the ratio of Lys:OL=1:1 showed typical CTE luminescent material with characteristic concentration-dependent luminescence and wave-

dependent luminescence, which is attributed to its heterogeneous vesicle aggregation structure in solution. 0% and 4.56% are the quantum yields at 0.1 mmol and 50 mmol, respectively. (Supplementary Figure 4 and Supplementary Figure 5 respectively support this conclusion in terms of fluorescence properties and aggregate structure)

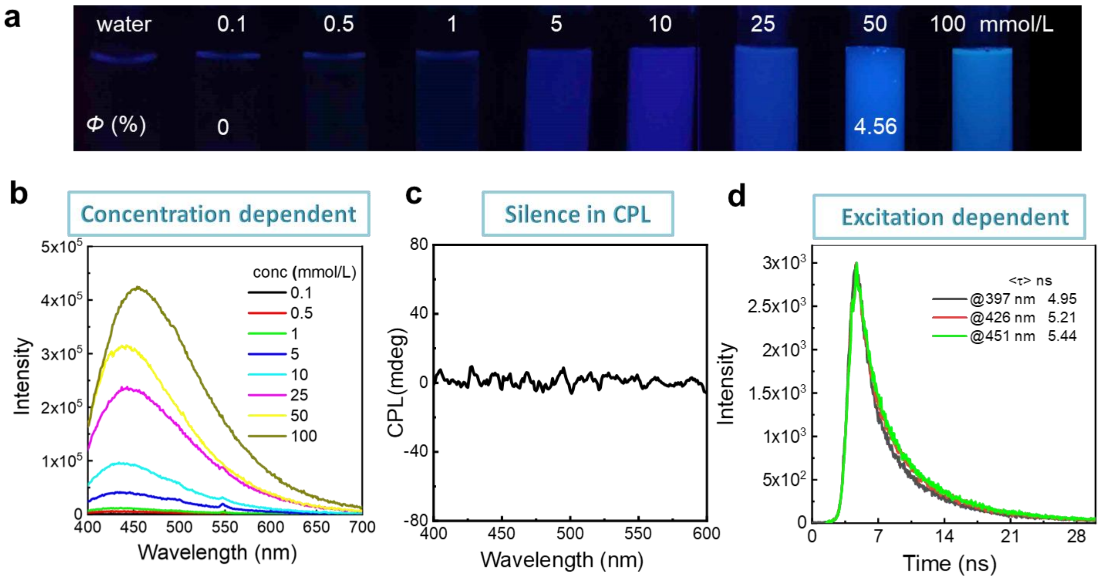

Supplementary Figure 4. The fluorescence properties of PLL-OL aqueous solutions. a Photographs taken under 365 nm UV light and **b** emission spectra (λ_{ex} = 365 nm) of PLL-OL aqueous solutions at different concentrations (Lys:OL=1:1). **c** CPL spectra (λ_{ex} = 350 nm) of PLL (50 mM, 1:1) aqueous solutions. **d** Lifetimes of PLL-OL (50 mM, 1:1) aqueous solutions monitored at 397(λ_{ex} = 310 nm), 426(λ_{ex} = 360 nm) and 451 nm (λ_{ex} = 390 nm).

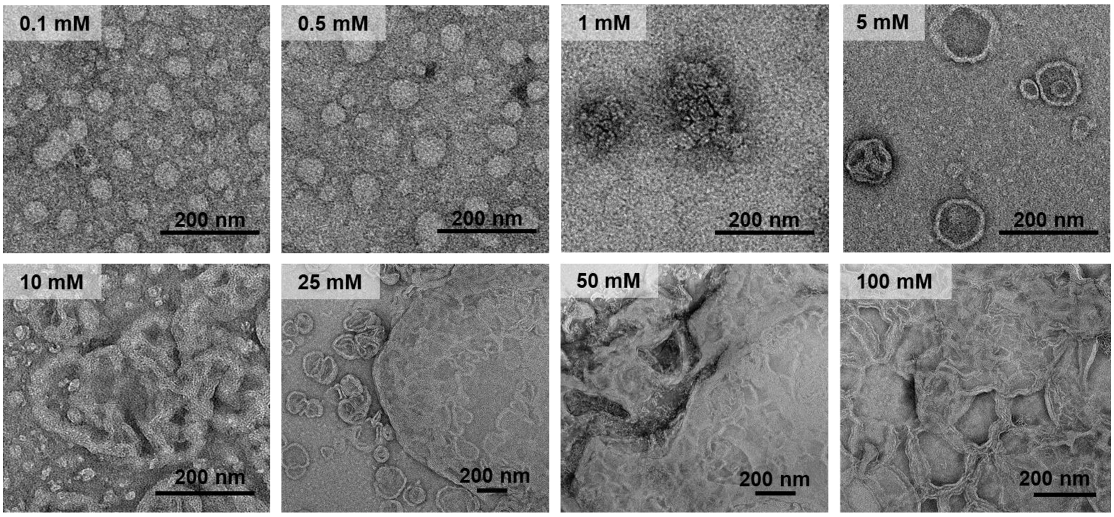

Supplementary Figure 5. TEM photos of PLL-OL aqueous solutions taken at different concentrations. (Lys:OL=1:1)

2.3 The composition and structure characterization of PLL-OL film

Supplementary Table 1. Table of Elemental Analysis of PLL-OL film

Element	N	C	H
Experimental result	5.12	68.61	11.31
Film composition	$(C_6H_{14}N_2O)_1 \cdot (C_{18}H_{34}O_2)_{1.405} \cdot (H_2O)_{1.267}$		

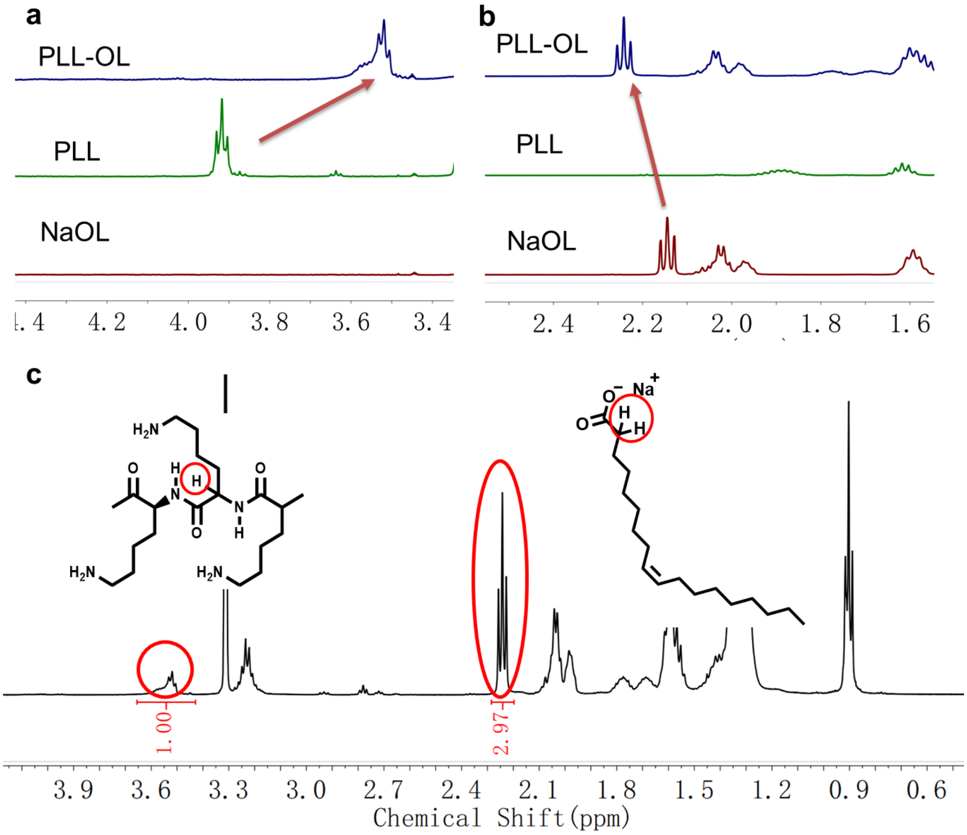

Supplementary Figure 6. ^{1}H-NMR spectra of PLL, NaOL and PLL-OL. The α-H of the OL carboxyl group is shifted from 2.14 ppm in NaOL to 2.24 ppm in the PLL-OL film(fig b). The α-H of the amide bond of PLL is shifted from 3.92 ppm in PLL powder to 3.52 ppm in the PLL-OL film(fig a).. The ratio of the peak area of these two hydrogens in PLL-OL film is 2.97:1(fig c), so the ratio of OL to Lys is 1.5:1.

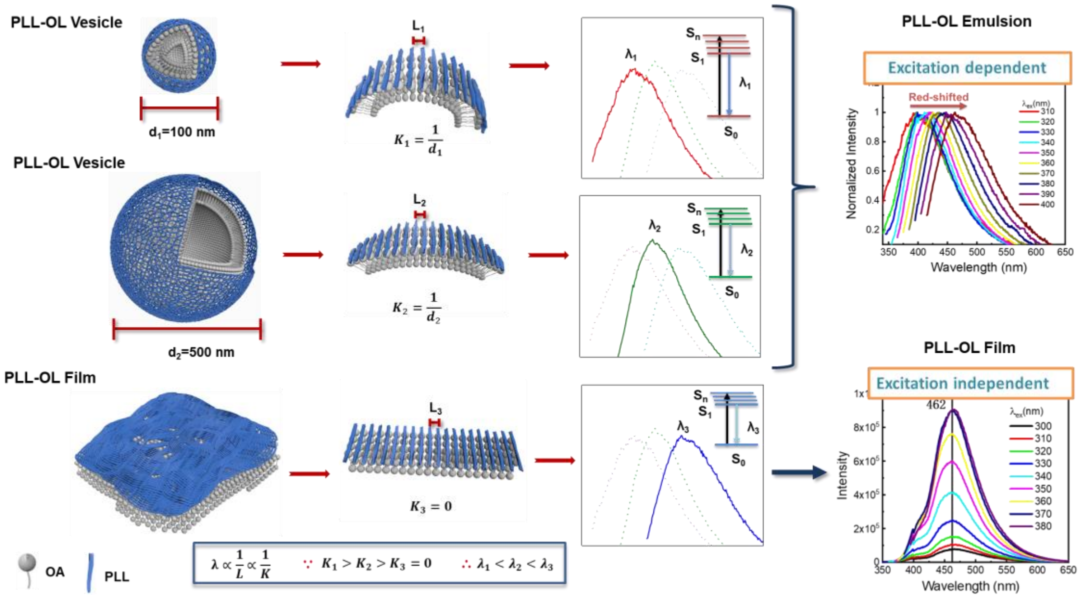

Supplementary Figure 7. The schematic diagram of the excitation-wavelength-dependent luminescence of PLL-OL in water and the non-excitation-wavelength-dependent luminescence of PLL-OL film. According to through-space-conjugation (TSC) theory, the spatial arrangement of electron-rich groups determines the luminescence of resulting from the clustered chromophores. In water, PLL-OL vesicles of different sizes are fromed. The larger the diameter(d) of the vesicle is, the smaller the curvature(K) is, which results in larger distance between the clusterolumingens. This means the through-space-conjugation (TSC) for large vesicles is better than for small vesicles. As a result, longer exictation wavelength is required for the large vesicles, and the system displays excitation dependent emission wavelength. However, in the PLL-OL films, all the vesicles transform into a planar stacked bilayer structure, the curvature of the system is 0, and the clustered chromophores are uniformly arranged in the entire film, thus forming a uniform spatial conjugation structure, which has optimum excitation

corresponding to the spatial conjugation. As a result, it shows similar luminescence properties to traditional chromophores.

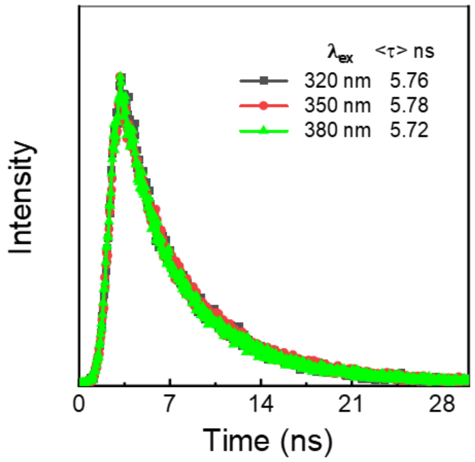

Supplementary Figure 8. Lifetimes of PLL-OL film monitored at 462 nm with different excitation wavelength.

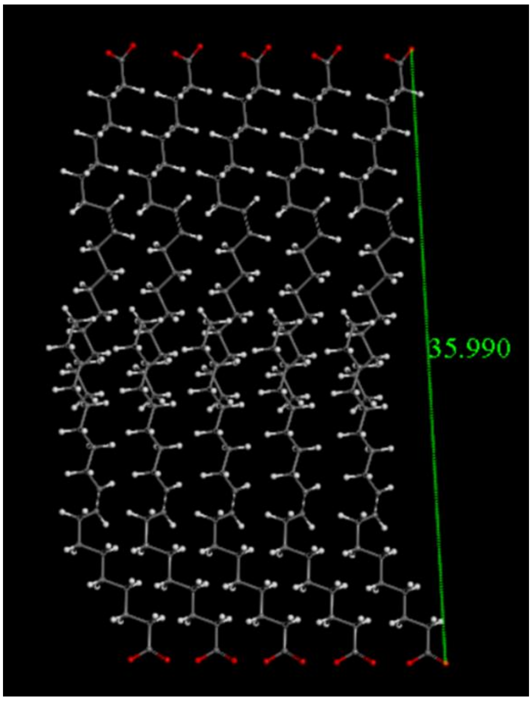

Supplementary Figure 9. The molecular simulation of oleate bilayer

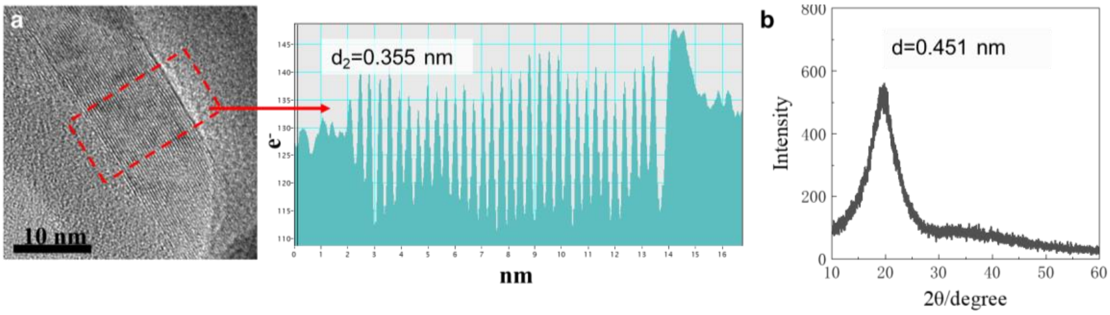

Supplementary Figure 10. Structure characterization of PLL-OL film. **a** TEM contrast analysis spectra shows that the interval between light and dark stripes is 0.355 nm. **b** Wide angle XRD patterns of PLL-OL film.

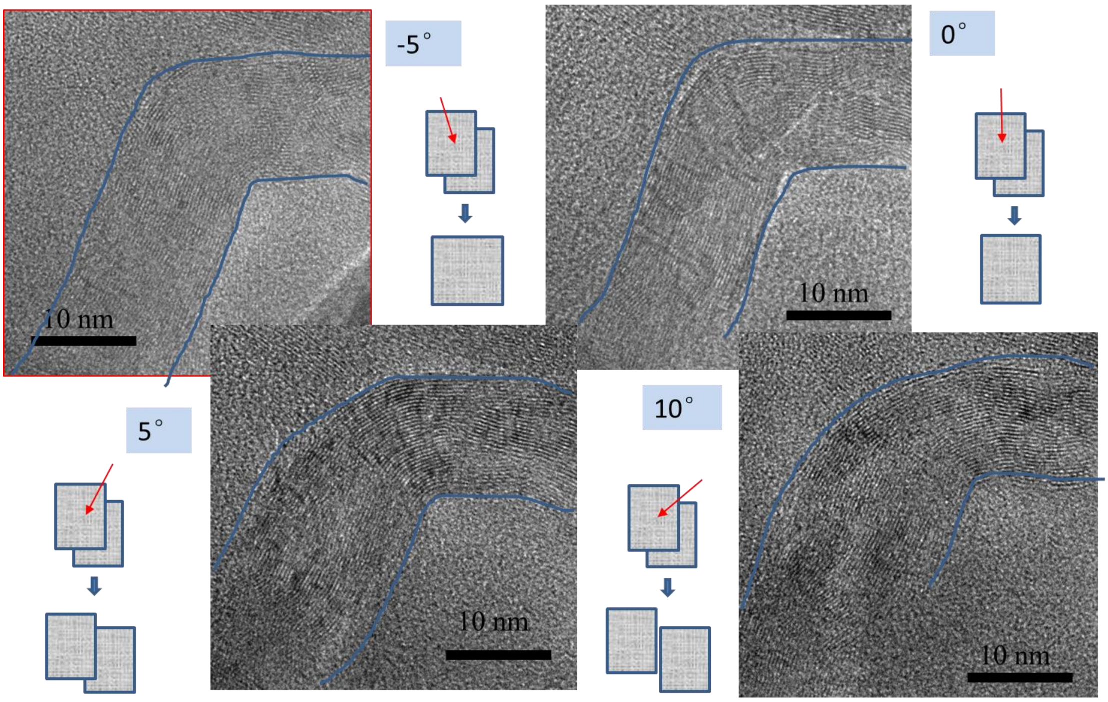

Supplementary Figure 11. Variation of the stripes in the PLL-OL film under TEM upon variation of the sample stage from -5 to 10°. The region with stripes becomes larger as the sample stage was tilted, indicating the overall visible area becomes larger due to the showing up of the sub layers.

2.4 Supplementary for the humidity responsive CTE-based CPL

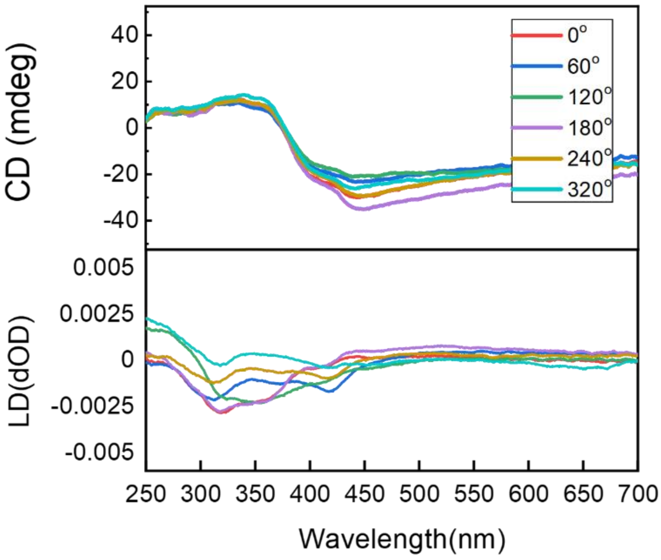

Supplementary Figure 12. The CD and LD spectra of PLL-OL film measured with different angles.

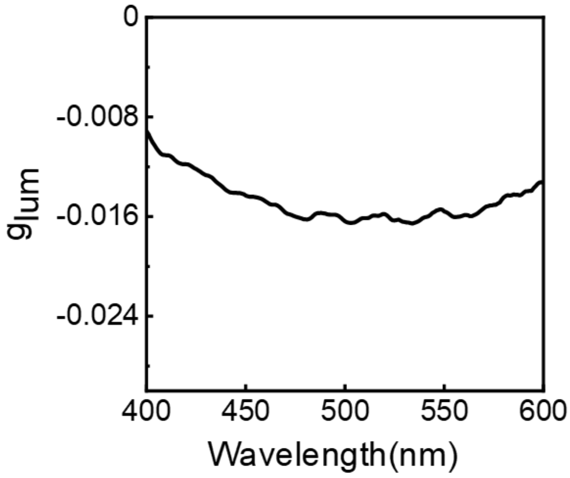

Supplementary Figure 13. The g_{lum} of PLL-OL film.

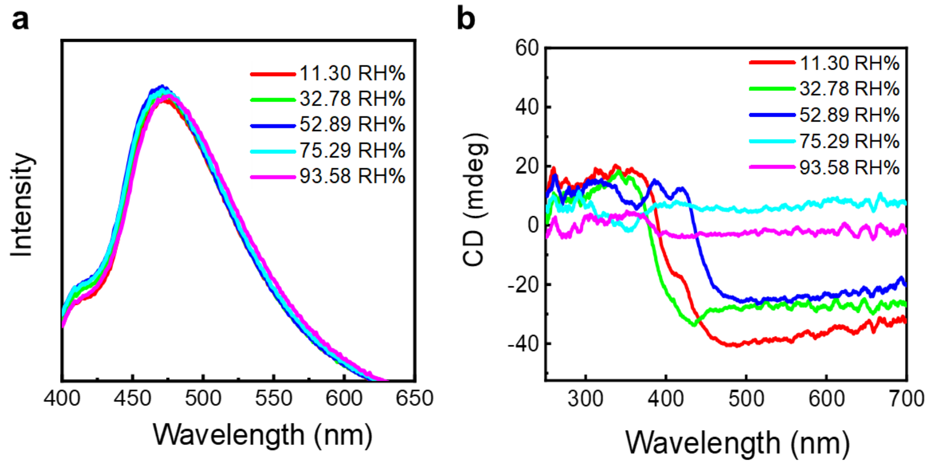

Supplementary Figure 14. The emission and CD spectra of PLL-OL films under different relative humidity. a Emission spectra; **b** CD spectra. The emission maximum slightly red-shifts as the humidity is as high as 93.58%, indicating that the folding state of the PLL chains has been changed in high water content.

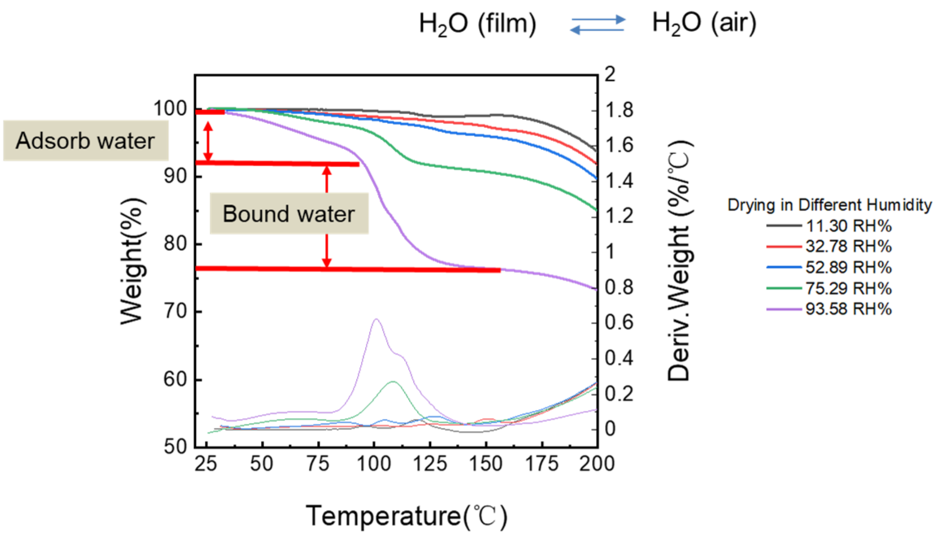

Supplementary Figure 15. TGA spectra of PLL-OL films under different relative humidity. The absorbed water will gradually evaporate with the increase of temperature, while the bound water will break the bond above 100°C and form an obvious peak on the TGA

Supplementary Table 2. The variation of interlayer spacing, water content and hydrogen bonding states in the film with variation of humidity.

RH %	2θ/degree	d/nm	Adsorb water (%)	Bound water(%)	υ_1 N-H(cm^{-1})	υ_2 N-H(cm^{-1})
11.30	2.58	3.42	0.5	0.4	3229	3402
32.78	2.44	3.62	2.3	1.0	3233	3405
52.89	2.3	3.84	2.5	1.5	3250	3442
75.29	2.02	4.37	3.0	5.5	3258	3484
93.58	2	4.42	6.4	16.9	\	\

2.5 Supplementary for the Color tunable CPL through FRET

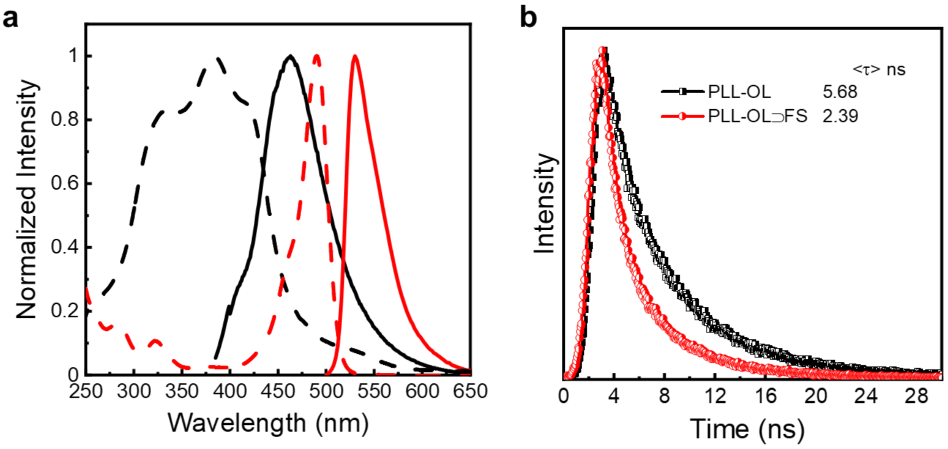

Supplementary Figure 16. The FRET from PLL to FS. a Normalized absorption (dashed line) and emission (solid line) spectra of PLL-OL film (black lines) and FS aqueous solution (red lines, 10^{-4} M). **b** Lifetimes of PLL-OL film (black) and PLL-OL⊃FS film (0.87 wt% FS, red) at RT.

Supplementary Table 3. Florescence lifetime, energy transfer efficiency and absolute quantum yield of the PLL-OL⊃FS film with different FS weight content

FS weight content	Fluorescence lifetime(ns)	Energy transfer efficiency(%)	Absolute quantum yield(%)
0 wt%	5.68		5.56
0.01111 wt%	4.38	22.9	7.89
0.1697 wt%	3.33	41.4	13.7
0.2727 wt%	2.49	56.2	19.2
0.4286 wt%	2.42	57.4	20.8
0.8727 wt%	2.39	57.9	21.9
1.091 wt%	2.4	57.7	19.5

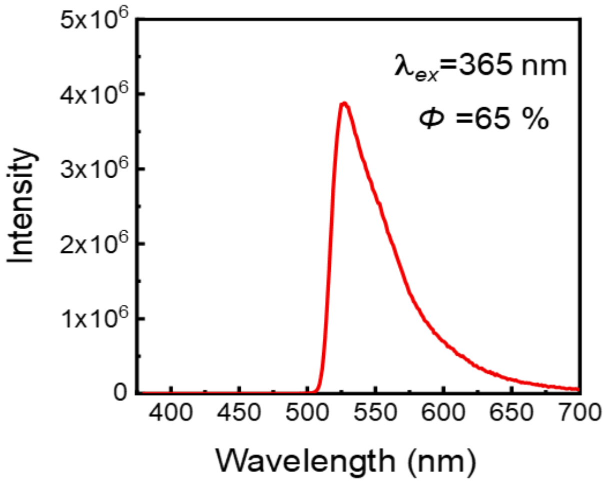

Supplementary Figure 17. The emission spectrum of 400 μM FS aqueous solution. (λ_{ex} = 365 nm)

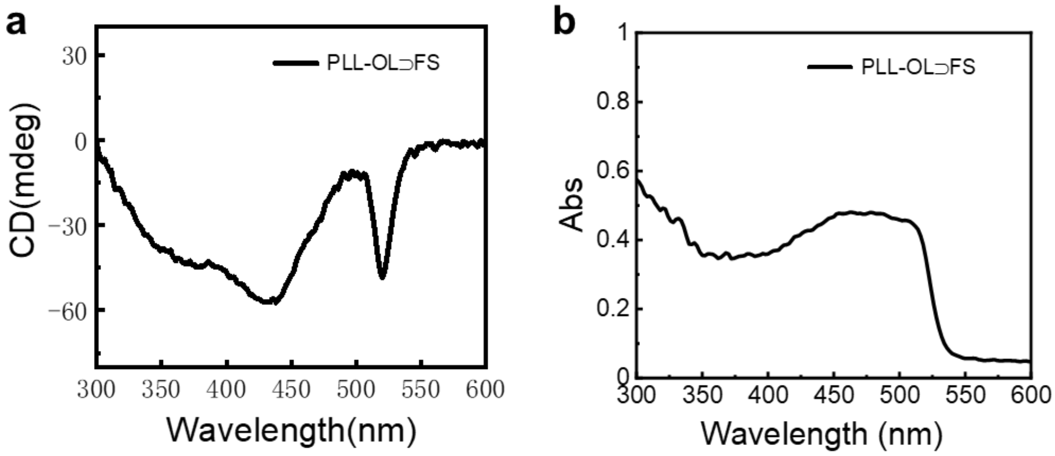

Supplementary Figure 18. The CD and UV spectra of PLL-OL⊐FS film (0.87 wt% FS). a CD spectra. **b** UV spectra.

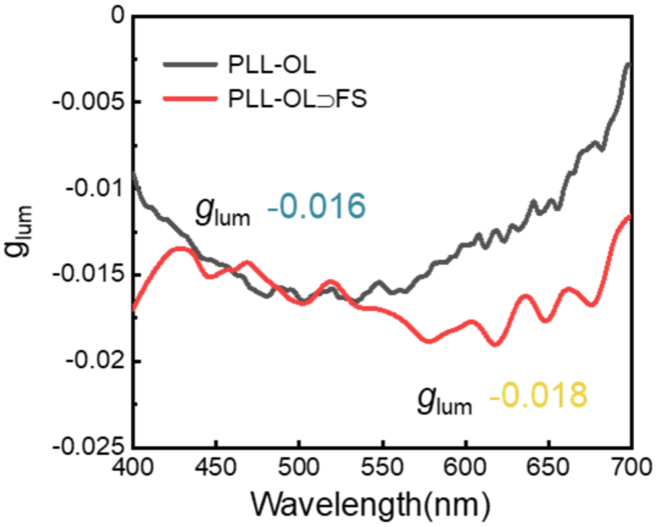

Supplementary Figure 19. The g_{lum} value of PLL-OL film and PLL-OL⊐FS film (0.87 wt% FS).

2.6 Control experiment

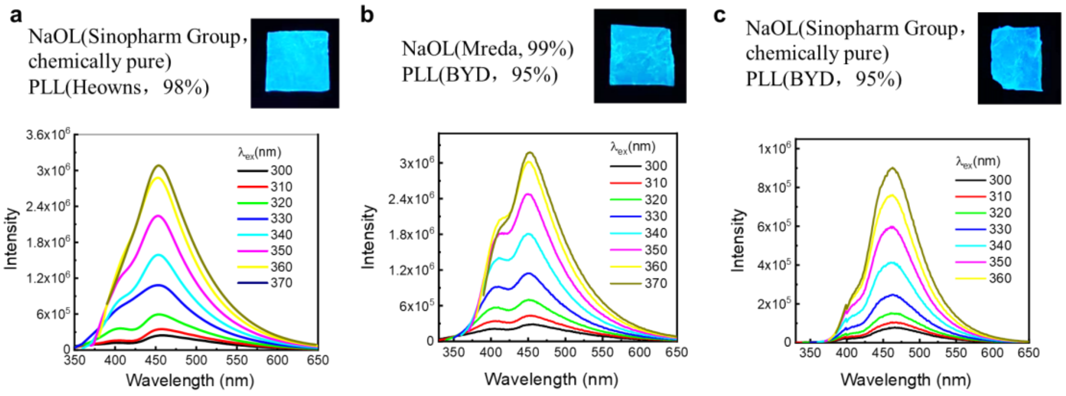

Supplementary Figure 20. The photos and emission spectra of PLL-OL films with PLL and NaOL of different sources and purity. a NaOL (Sinopharm Group, chemically pure) and PLL (Heowns, 98%) **b** NaOL (Mreda, 99%) and PLL (BYD, 95%) **c** NaOL (Sinopharm Group, chemically pure) and PLL (BYD, 95%).

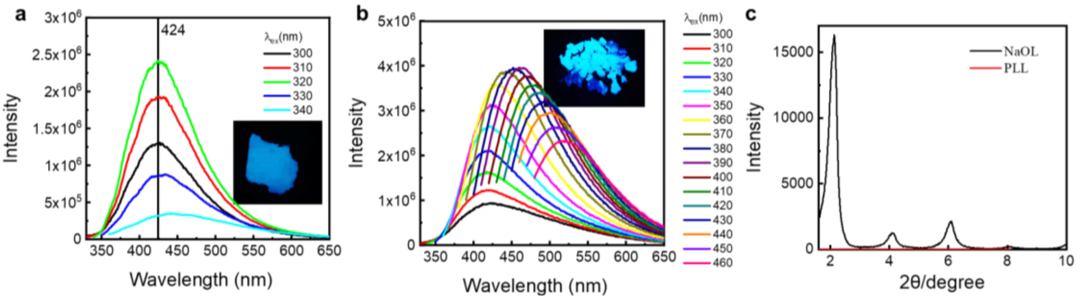

Supplementary Figure 21. The spectra and structure information of NaOL and PLL in solid state. **a** Emission spectra of neat NaOL at different excitation wavelengths. **b** Emission spectra of neat PLL at different excitation wavelengths. **c** XRD patterns of neat NaOL (black) and neat PLL (red).

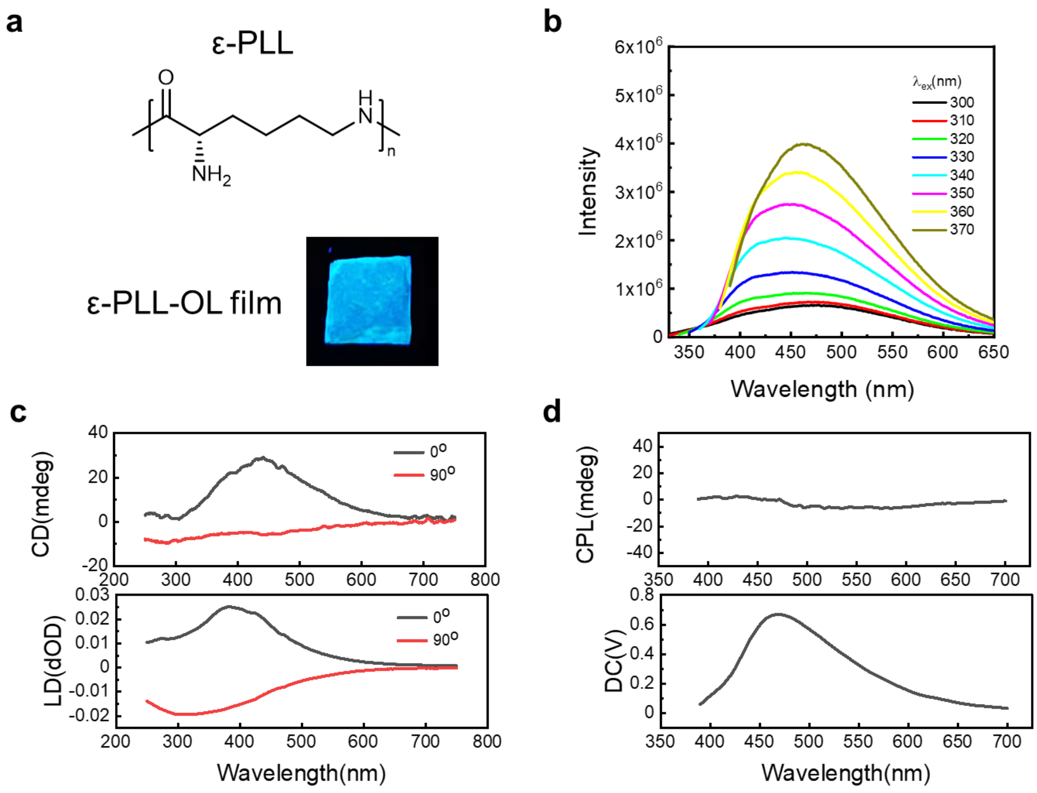

Supplementary Figure 22. **The emission, CD and CPL spectra of ε-PLL-OL film. a** the structure of ε-PLL **b** Emission spectra of ε-PLL-OL film at different excitation wavelengths. **c** CD spectra. **d** CPL spectra.
